# Attempted suicidal poisoning with pretilachlor: case series of a herbicide simulating organophosphorus toxicity

**DOI:** 10.1093/omcr/omad139

**Published:** 2023-12-19

**Authors:** Agnimshwor Dahal, Sudeep Shukla, Bhim Chauhan, Jeevan Gyawali, Astha Thapa, Bishal Budhamagar, Deergha Mishra

**Affiliations:** Nepal Intensive Care Research Foundation, Kathmandu, Nepal; Department of Emergency Medicine, Nepalgunj Medical College Teaching Hospital, Kohalpur, Nepal; Patan Academy of Health Sciences - School of Medicine (PAHS-SOM), Lalitpur, Nepal; Patan Academy of Health Sciences - School of Medicine (PAHS-SOM), Lalitpur, Nepal; Patan Academy of Health Sciences - School of Medicine (PAHS-SOM), Lalitpur, Nepal; Department of Emergency Medicine, Nepalgunj Medical College Teaching Hospital, Kohalpur, Nepal; Department of Emergency Medicine, Nepalgunj Medical College Teaching Hospital, Kohalpur, Nepal

## Abstract

Pretilachlor is a herbicide that can cause clinical symptoms in people that are comparable to those of organophosphate poisoning when ingested. Given how closely it mimics the toxicity of organophosphate compounds, it presents a significant challenge to clinicians during management. The following cases were presented to the Emergency Department at Nepalgunj Medical College Teaching Hospital, Kohalpur, Banke, Nepal.

## INTRODUCTION

Ingestion of pretilachlor, a synthetic chloroacetanilide herbicide, is not commonly observed, despite its widespread use in the agricultural regions of the Indian subcontinent [1]. Chloroacetanilide herbicides are a class of anilide herbicides, a subclass of amide herbicides. These include pretilachlor, butachlor, s-metolachlor, acetochlor, and dimethachlor. Pretilachlor is a selective and broad-spectrum herbicide. The compound is known by the name 2-chloro-2′,6 diethyl-N-(2-propoxyethanol) acetanilide. In rice and paddy fields, it is used to get rid of practically all kinds of weeds, including annual grasses, sedges, broad-leaved weeds, and floating aquatic species [2]. In comparison to other herbicides, it is less harmful to crops. It has been discovered that it works by preventing the formation of lipids, proteins, fatty acids, alcohol, and isoprenoids, among other things. The prolonged use of these herbicides has likely cancer-causing consequences [3]. Pretilachlor’s acute toxicity after ingestion can be mistaken for organophosphate pesticide toxicity, which can lead to improper patient management because these patients frequently exhibit the signs and symptoms of organophosphate toxicity, such as lacrimation, nausea, vomiting, and bladder and bowel incontinence [4]. Tachycardia, hypotension, sweating, and muscular spasms are seen. By treating symptoms, patients frequently make a full recovery without the use of atropine and oximes [5]. Here, we present a report of six cases of attempted suicide poisoning with pretilachlor herbicide and related organophosphorus toxicity symptoms.

## CASE SERIES

1. A 38 years old female presented to the emergency room with the alleged history of suicidal ingestion of unknown poison 1 h back following an argument with her husband. On presentation, the patient had 2 episodes of vomiting in the ER with excessive salivation, lacrimation, and dizziness. According to her family members, she was found unconscious with an empty container of pretilachlor 50% emulsified concentrate (EC) next to her. They noticed that about 250 ml of the substance had been used from the bottle (2 g/kg). She did not have any chest pain or shortness of breath. There was no previous history of suicidal attempts. On examination, the patient was drowsy with Glasgow Coma Scale (GCS) of 12/15 (E3 V4 M5). She was found to have tachycardia with a radial pulse of 101 beats per minute (BPM) and a blood pressure (BP) of 110/80 mmHg. She was afebrile with an oxygen saturation of 98% in room air and a respiratory rate (RR) of 19 breaths/min. The garlicky odor of organophosphate was not present on her breath. Other systemic examinations were unremarkable.
2. A 25-year-old male was referred by a primary center to our hospital with an alleged history of ingestion of pretilachlor 50% EC around 200 ml (2.2 g/kg) 3 h back. On arrival, the patient presented with complaints of nausea, multiple episodes of vomiting, and abdominal pain. The patient was semiconscious in the state with profuse lacrimation and sweating. He had urinary incontinence; however, bowel function was normal and no shortness of breath was noted. Upon examination, he was found to have a radial pulse of 66/min with blood pressure 100/70 mmHg, respiratory rate of 16 breaths/min, and oxygen saturation at 98% with no raised temperature. The remaining systemic examination was unremarkable.
3. A 21-year-old female presented to the emergency room with a history of ingestion of pretilachlor 50% EC herbicide 2 h back (2.8 g/kg). Her attendant had brought a 250 ml bottle of the same herbicide which she allegedly ingested. She complained of abdominal pain, lacrimation, multiple episodes of vomiting and salivation. Her vitals were; BP: 120/80 mmHg, pulse of 101 BPM, RR of 21/min, afebrile and Spo2 99% in room air. She was ill looking yet oriented to time, place and person with unremarkable systemic examinations. No peculiar garlicky smell was found in her breath.
4. A 59 years old male presented to the emergency room with the alleged history of suicidal ingestion of an unknown poison under the influence of alcohol after a family dispute which was later found to be pretilachlor 50% EC approximately 250 ml in amount (1.9 g/kg). Upon presentation he had mild abdominal cramps, vomiting, frothing from mouth and involuntary passage of urine. He had no known comorbidities. His vitals revealed normal body temperature, blood pressure of 130/90 mmHg, radial pulse 99 beats/min, respiratory rate of 18 breaths/min. His oxygen saturation was at 97%. His systemic examination was found to be unremarkable. No garlicky breath was detected but instead, he smelled of alcohol.
5. A 19 years old female presented to the emergency room with an alleged history of ingestion of ‘Hi-fit herbicide’ approximately 200 ml, the constituents of which turned out to be pretilachlor 50% EC (2.5 g/kg). Upon presentation she had shortness of breath, vomiting and chest pain. Additionally, there were no frothing or abnormal body movements. Her bowel and bladder habit had no change. Her vitals included a blood pressure of 100/70 mmHg, pulse 102 beats/min, oxygen saturation of 99% at room air and a respiratory rate of 18 breaths/min. In addition to this her systemic examination turned out to be unremarkable.
6. A 24 years old female presented to the emergency room with the alleged history of ingestion of pretilachlor 50% EC approximately 100 ml (1 g/kg). She complained of epigastric burn and nausea following ingestion of the poison. The vitals of the patient upon arrival were a blood pressure of 100/80 mmHg, pulse of 109 beats/min, oxygen saturation of 98% and respiratory rate of 23 breaths/min. The systemic examination was within normal limits.

All the above patients confirmed to have ingested pretilachlor 50% emulsified concentrate (EC). Upon presentation they were all subjected to gastric lavage as they all presented within 3 h of time frame. The vital signs were stable and were carefully monitored while being managed with supportive measures such as intravenous fluids, antiemetics and prophylactic antibiotics. The patients were kept nil per oral (NPO). Their vital signs remained steady, and normal urine production was ensured as a result. Later, the early symptoms of nausea, vomiting, salivation, lacrimation, and abdominal cramps disappeared.

In all the patients serial blood tests were performed including total blood counts, random blood glucose, liver and renal function tests, the reports of which turned out to be within normal ranges. Serum cholinesterase levels were also examined in a few cases, although they did not exhibit any abrupt changes in levels.

The patients were all subjected to close hemodynamic monitoring which turned out to be uneventful. They were kept under observation for a few days and later discharged following a psychological assessment and behavioral therapy. The subsequent visits turned out to be unremarkable.

## DISCUSSION

Pretilachlor, a synthetic chloroacetanilide herbicide, is used to kill practically all weed species in rice and paddy crops, including annual grasses, sedges (grass-like weeds), broad-leaved weeds, and floating aquatic plants. Marketed as a 50% EC, pretilachlor, it is a liquid that has no color or smell. It has alkyl aryl sulfonate of calcium salt as a surfactant and ethoxylated vegetable oil as an emulsifier [2]. We have presented six confirmed cases of pretilachlor ingestion and treated in line with OP poisoning. This is not uncommon in our region, as patients often treated for OP poisoning actually ingest chloroacetanilide products with symptoms similar to those of OP poisoning [4]. While OP poisoning is commonly encountered in the ER and is one of the leading unnatural causes (suicide) of death in Nepal [6], very few studies have reported pretilachlor poisoning in our part of the world [1, 4]. Despite this, accurate incidence has not been measured.

The chloroacetanilide class of herbicides is postulated to cause cytotoxicity by depleting GSH content in a dose-dependent manner in hepatocytes. In addition, it is also found that their mechanism appears to be reactive oxygen species generation, mitochondrial dysfunction and disruption in the cell cycle regulation [3]. In certain investigations, chronic exposure to chloroacetanilide has been linked to genotoxicity, carcinogenicity, and neurotoxicity [7]. Although such situations have only sometimes been documented to date, acute oral exposure may have different effects on people. Despite its modest toxicity, a retrospective investigation of 35 individuals with acute intake of the substance led to the death of one patient and the unconsciousness of three others within 24 h [5]. Another study was carried out by Lo et al in 113 patients who had been exposed orally to chloroacetanilide like butachlor and alachlor, and it found that one-fourth of the patients were asymptomatic, the rest had vomiting and neurological symptoms like drowsiness and central nervous depression, along with three deaths from profound hypotension and coma [8].

Patients with pretilachlor poisoning usually present with clinical symptoms like nausea, vomiting, diarrhea, drowsiness, stupor, seizures, and fever which can closely mimic cholinergic toxicity. However, peculiar characteristics of organophosphate poisoning including miosis, garlicky smell, bradycardia, lacrimation, and salivation are typically not seen [5, 8].

In our instances, patients commonly present with symptoms of nausea, vomiting, abdominal pain, and tachycardia. Signs like fever, bradycardia, miosis, and hypotension were not present in any patients.

Below is a table illustrating the common signs and symptoms observed in our patients on initial presentation to the emerg0ency room:

**Table:** 

Clinical features	Number of patients
**Symptoms**	
Drowsiness	1
Nausea	5
Vomiting	5
Abdominal pain	3
Diarrhea	0
Difficulty breathing	1
Chest pain	1
Increased urination	0
Drowsiness	0
Seizure	0
**Signs**	
Bradycardia	0
Tachycardia	4
Hypotension	0
Fever	0
Tachypnea	1
Miosis	0
GCS < 15/15	1

On initial presentation, serum acetylcholinesterase enzyme level was checked in all patients as it was difficult to differentiate pretilachlor poisoning from OP poisoning based on clinical presentation alone. OP poisoning can be detected by measuring acetylcholinesterase (AchE) level which is decreased [9] whereas it is within the normal range in pretilachlor poisoning [10]. In all our patients, the enzyme level came out to be normal, which was a major hint to finally differentiate between the two poisonings.

To offset the cholinergic symptoms of OP poisoning, atropine is administered and pralidoxime is given as an antidote which reverses the permanent binding and disintegration of AChE by OP There is no antidote for pretilachlor poisoning [10] unlike OP poisoning where atropine is administered to offset the cholinergic symptoms and pralidoxime is given as an antidote which reverses the permanent binding and disintegration of AChE by OP [9]. The majority of treatment for pretilachlor poisoning is symptomatic care, which includes stabilizing the patient, fluid resuscitation, and vigilant hemodynamic monitoring [5]. Skin decontamination with soap and water as well as GI decontamination is the same in both the poisonings [9]. In all the cases, airway, breathing, and circulation were assessed and simultaneously maintained immediately. Since all the patients presented within 3 h of ingestion and had normal GCS, gastric lavage was done immediately.

All of the patients were admitted to the medical ward, where they received IV fluids, and proton pump inhibitors, and were kept nil by mouth (NPO). Urinary output and hemodynamic condition were continuously recorded. Because pretilachlor and its class of herbicides are known to be hepatotoxic and increase liver enzymes [10], liver function tests (LFTs) were monitored in all patients and were normal. In addition, patients with pretilachlor poisoning are more likely to develop rhabdomyolysis, thus patients’ renal function tests (RFT) and serum electrolytes were additionally monitored [5]. The investigations stated above were also within the normal range. Behavioral evaluation and psychiatric consultation were conducted during hospital admission. They were discharged after 3 days of hospital admission as all of them were clinically stable. On follow-up after one week, no clinical signs and symptoms were observed.

While all our cases were stable and recovered completely, there are instances of patients requiring critical care and also fatalities. Factors like old age, lower bicarbonate levels, presence of CNS effects [5], oral ingestion, a higher dose of ingestion, and hypotension [8] have been observed to be associated with poor prognosis.

## CONCLUSION

These instances highlight the fact that, in spite of exhibiting clinical characteristics like those of OP toxicity, pretilachlor poisoning is differentiated from OP poisoning on the basis of absence of typical cholinergic clinical signs and symptoms and presence of normal AChE level. Immediate stabilization, attentive monitoring, and symptomatic treatment are the three crucial elements from the recovery of pretilachlor toxicity. Clinicians must have a thorough understanding of such herbicides in order to manage patients effectively.
